# Autologous genome-edited hematopoietic stem cells correct Gaucher disease and establish a platform for clinical translation

**DOI:** 10.21203/rs.3.rs-7123212/v1

**Published:** 2025-08-18

**Authors:** Natalia Gomez-Ospina, Luisa Pimentel Vera, Marc Gastou, Pasqualina Colella, Jessica Arozqueta Basurto, Allan Feng, Yi Lin, Xueheng Zhao, Ying Sun

**Affiliations:** Stanford University; Stanford University; University of California, San Francisco,; Stanford University; Stanford University; Stanford University; Cincinnati Children’s Hospital Medical Center; Cincinnati Children’s Hospital Medical Center; Cincinnati Children’s Hospital Medical Center

**Keywords:** Gaucher, transplantation, hematopoietic, genome editing, CRISPR, busulfan, irradiation

## Abstract

Gaucher disease type 1 is a lysosomal storage disorder caused by *GBA1* mutations that reduce glucocerebrosidase activity, leading to glycolipid buildup, particularly in macrophages. To develop a curative approach, we established a high-efficiency genome editing platform for human and murine hematopoietic stem-progenitor cells using CRISPR/Cas9, recombinant adeno-associated virus serotype 6. To enhance homology-directed DNA repair while minimizing genotoxicity, we incorporated a new 53BP1 inhibitor, a ubiquitin variant that promotes DNA end resection and significantly increases editing efficiency. This enabled precise insertion of a human *GBA1* transgene—driven by a macrophage-specific promoter—into the mouse *Rosa26* and human *CCR5* safe-harbor loci. To assess efficacy, we established a rapidly progressive Gaucher disease mouse model by inducing hematopoietic-specific *Gba1* deletion in a D427V background. Transplantation of edited cells corrected hematologic and visceral abnormalities, normalized lipid storage, and was effective under myeloablative and reduced-intensity busulfan conditioning. Notably, therapeutic benefit was achieved with only ~ 3% edited allele engraftment. These findings offer strong proof-of-concept for ex vivo genome editing as a mutation-agnostic, potentially curative strategy for Gaucher disease and support its clinical advancement.

## Introduction

Lysosomal storage diseases (LSDs) are a heterogeneous group of inherited metabolic disorders characterized by the accumulation of undegraded macromolecules within lysosomes, resulting from deficiencies in specific lysosomal enzymes or transport proteins. Among these, Gaucher disease (GD) is one of the most common and arises due to biallelic pathogenic variants in the *GBA1* gene, which encodes the lysosomal enzyme glucocerebrosidase (GCase) [1]. When this enzyme is deficient, glucocerebrosides accumulate within various cells but primarily in those of the monocyte-macrophage lineage [2].

Gaucher disease (GD) presents as a spectrum of clinical severity; however, for clinical management and prognostic purposes, it is traditionally classified into three main types based on the presence or absence of neurological involvement. GD type 1 (GD1) is the most common form in Western populations, accounting for approximately 94% of cases [3, 4]. In GD1, glucocerebroside-laden macrophages, known as Gaucher cells, infiltrate various organs, leading to progressive hepatosplenomegaly, anemia, thrombocytopenia, bone disease, and, in some cases, pulmonary involvement. Patients with GD1 also exhibit an elevated lifetime risk of multiple myeloma and Parkinson’s disease [5, 6].

Current standard-of-care therapies for GD1 include enzyme replacement therapy (ERT) and substrate reduction therapy (SRT) [4]. Both treatments necessitate lifelong administration [7–10]. ERT involves biweekly intravenous infusions of recombinant GCase targeted to macrophages, while SRT uses oral inhibitors of glucosylceramide synthase to reduce substrate accumulation. These therapies effectively manage visceral and hematologic disease but fail to completely address pulmonary and skeletal complications, and neither crosses the blood-brain barrier to treat neuronopathic forms [11–14].

Additionally, both modalities require lifelong administration and impose substantial financial burdens, with annual costs ranging from $300,000 to $450,000 and lifetime costs up to $22 million per patient, limiting its availability worldwide [15, 16]. Thus, while ERT and SRT can mitigate certain clinical features of GD1, they are non-curative and provide no therapeutic benefit for neuronopathic GD.

Allogeneic hematopoietic stem cell transplantation (allo-HSCT) has been successfully employed as a one-time treatment for GD1 and is sometimes used for GD3. Its therapeutic benefit arises from the durable engraftment of donor-derived, glucocerebrosidase-competent hematopoietic cells, which give rise to macrophages capable of degrading glucocerebroside and functioning as a long-term enzyme depot [17–23]. In the CNS, bone marrow-derived cells derived from the graft can migrate across the blood-brain barrier, differentiate into microglia-like cells, and provide enzymatic cross-correction to neurons and glia, and attenuate neuroinflammation [24, 25]. Despite these benefits, allo-HSCT is largely restricted to resource-limited settings or to patients with refractory disease, as it carries substantial risks, including graft-versus-host disease, transplant-related morbidity, and the need for suitable donor matches [18, 19, 26, 27]. Nonetheless, compared to ERT and SRT, allo-HSCT offers distinct advantages, including the potential for lifelong correction of systemic disease, and a potential benefit in neuronopathic GD [27–31].

Given the potential for HSCT to constitute a one-time therapy for GD1, improving its safety would be significant. An autologous approach could eliminate the morbidity of graft-versus-host disease, speed up hematopoietic reconstitution, reduce the risks of immunosuppression, and lead to earlier intervention by obviating the need for donor search/matching. Genome editing provides a powerful strategy for generating autologous gene-modified hematopoietic products. The CRISPR/Cas9 system, originally derived from bacterial adaptive immunity, enables precise, site-specific DNA cleavage through a programmable single-guide RNA (sgRNA) [32]. Following double-strand breaks, DNA repair proceeds via either non-homologous end joining (NHEJ), which introduces small insertions or deletions, or homology-directed repair (HDR), which facilitates precise sequence insertion or replacement when a repair template is supplied. Replacing entire coding sequences using HDR-based repair is particularly well-suited for disorders with extensive allelic heterogeneity, such as GD, for which more than 500 pathogenic mutations have been identified [33]. Instead of designing mutation-specific strategies, a universal approach can be implemented by inserting an expression cassette into a non-essential genomic “safe harbor” locus.

We and others have developed and optimized a homology-directed repair (HDR)-mediated genome editing strategy in hematopoietic stem and progenitor cells (HSPCs) using a ribonucleoprotein (RNP) complex composed of Cas9 and sgRNA, along with a recombinant adeno-associated virus serotype 6 (rAAV6) to deliver the HDR template [34–37]. Leveraging this RNP-rAAV6 system, we successfully inserted a functional *GBA1* coding sequence driven by a monocyte/macrophage-specific promoter (CD68S) into the *CCR5* safe harbor locus in human HSPCs [35]. This platform enables a mutation-independent correction strategy applicable to all patients with GD1, regardless of their specific *GBA1* mutations. *CCR5*, which encodes a co-receptor for HIV-1, is considered non-essential—individuals homozygous for the CCR5Δ32 deletion lack functional CCR5 and remain healthy while exhibiting resistance to HIV-1 infection [38]. Multiple studies, including our own, have validated CCR5 as a suitable safe harbor for stable, therapeutic transgene expression, supporting its use as a universal platform for genome editing in lysosomal storage disorders [36, 39, 40].

This study reports the preclinical efficacy of a therapeutic analog composed of murine HSPCs edited at the *Rosa26* safe harbor locus to express a human GCase from a monocyte-macrophage-specific cassette. To enable testing, we developed a genome-editing protocol using the RNP-rAAV6 platform and HDR enhancer molecules in mouse HSPCs, significantly improving editing efficiency. To rigorously evaluate therapeutic efficacy, we established a new, rapidly progressive mouse model of GD1 that more faithfully recapitulates the visceral, hematologic, and skeletal manifestations observed in patients. Using this model, we demonstrate that transplantation of genome-edited autologous HSPCs, following either total body irradiation or sub-myeloablative busulfan-based conditioning, restores GCase activity, reduces glucosylceramide and glucosylsphingosine accumulation, reverses hepatosplenomegaly, corrects anemia, and improves histopathology. Importantly, we identified a minimal effective dose of edited alleles in the bone marrow and GCase activity in the hematopoietic system that is sufficient to normalize disease parameters. To facilitate clinical translation, we established a genome editing protocol suitable for clinical applications that allow for high-efficiency, HDR-mediated integration of the GCase cassette into the CCR5 locus of adult human CD34^+^ HSPCs. The new protocol used an optimized gRNA and HDR enhancer, resulting in robust editing, preserving cell viability, and reducing rAAV doses by 10-fold. Together, these findings establish robust proof-of-concept for autologous HSPC gene editing as a curative strategy for GD1 and provide a compelling rationale for advancing this platform toward clinical translation.

## Results

### A new inducible mouse model for rapid-onset GD1

Previous GD1 mouse models were generated by conditionally knocking out GCase in the hematopoietic system using the Cre-loxP system [41, 42]. In these models, the *Gba1* gene was flanked by loxP sites at exons 9–11, and Cre recombinase expression was driven by the interferon-inducible Mx1 promoter. Administration of polyinosinic:polycytidylic acid (pIpC), a synthetic double-stranded RNA, triggered Cre expression and enabled temporal control of *Gba1* deletion in hematopoietic cells [43, 44]. While these models exhibited visceral features of GD by 12 months of age, hematologic and skeletal abnormalities developed slowly and often failed to fully recapitulate the clinical phenotype observed in human GD1 [41, 42].

**Figure 1:**
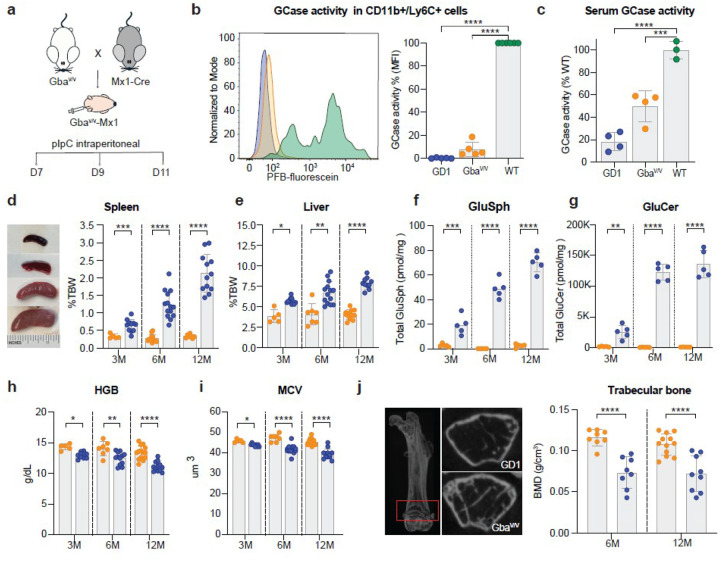
A new inducible mouse model for rapid-onset GD1 **a**, Schematic showing the generation of the Gba1-Mx1 mouse and post-natal induction of Cre using polyinosinic: polycytidylic acid (pIpC). Induction of Cre results in excision of exons 6–8 of the *Gba1* gene, leading to the visceral, hematologic, and bone phenotype. **b**, Representative histograms showing GCase activity in peripheral blood CD11b^+^/Ly6C^+^ cells, measured by flow cytometry using the membrane-permeable fluorogenic substrate PFB-FDGlu (5-(Pentafluorobenzoylamino) Fluorescein Di-beta-D-Glucopyranoside). GCase activity is reported as mean fluorescence intensity (MFI) of Pentafluorobenzoylamino)fluorescein (PFB-F) in mice of the following genotypes: 1) wild-type (Gba+/+ or WT, green, n=6), 2) homozygous Gba1^f/f;D427V/D427V^ lacking the Mx1-Cre transgene (Gba^V/V^, yellow, n=5), and 3) pIpC-injected Gba1^f/f;D427V/D427V^-Tg(Mx1-Cre) (GD1, blue, n=5). **c**, Serum GCase enzyme activity in the same mice (Gba+/+ n=4, Gba^V/V^ n=4, GD1 n=3). **d,** Spleen and liver weights as a percentage of total body weight for the same mice (Gba^V/V^, n=5 (3 months), 7 (6 months), and 13 (12 months), GD1 n=11 (3 months), 14 (6 months), and 12 (12 months)). **e,** Liver weights as a percentage of total body weight for the same mice (Gba^V/V^, n=5 (3 months), 7 (6 months), and 12 (12 months), GD1 n=11 (3 months), 14 (6 months), and 10 (12 months)). **f, g**, Hemoglobin (HGB) and mean red blood cell volumes (MCV) measured in blood for the same mice (Gba^V/V^, n=5 (3 months), 6 (6 months), and 8 (12 months), GD1 n=9 (3 months), 12 (6 months), and 12 (12 months)). **h, i**, Total liver Glucosylsphingosine (GlcSph) and glucosylceramide (GlcCer) levels expressed as picomoles of substrate per milligram of protein in the same mice at 3, 6, and 12 months of age (n=5 for all genotypes). **j**, Trabecular bone mineral density (BMD) in the distal femur was measured in Gba^V/V^ and GD1 mice at two time points: 6 months (n=8 per group) and 12 months (GbâV/V, n=12; GD1, n=8). **b-j**, Data are shown as Mean ± SD, **b-c,** One-way ANOVA with Dunnett’s multiple comparison test **d-j,** Two-way ANOVA with Sidak’s multiple comparison test. Significance levels indicated as *p < 0.05, **p < 0.01, ***p < 0.001, and ****p < 0.0001.

To model a more aggressive disease course, we employed the same Mx1-Cre system but used mice homozygous for a floxed *Gba1* allele bearing the D427V substitution (orthologous to the human D409V mutation), with loxP sites flanking exons 6–8. This configuration reduced residual GCase activity to 2–10% of wild-type levels in all tissues, limiting compensatory enzyme activity from non-hematopoietic tissues [45, 46]. We induced *Gba1* excision via three pIpC injections on postnatal days 7, 9, and 11 (Fig. 1a **and Supplementary Fig. 1**). Excision efficiency was quantified by droplet digital PCR (ddPCR) using a custom-designed assay that amplified across the novel exon 5–exon 9 junction, thereby specifically detecting only the excised *Gba1* allele. GD1 mice—defined as mice homozygous for the floxed D427V *Gba1* allele and carrying the Mx1-Cre transgene that were injected with pIpC (*Gba1*^f/f;D427V/D427V^-Tg(Mx1-Cre) + pIpC)—exhibited approximately 98% excision of *Gba1* alleles in bone marrow and peripheral blood, with recombination restricted to hematopoietic cells (**Supplementary Fig. 1**). Compared to homozygous mice lacking the Cre transgene (Gba^V/V^) and wild-type mice at the *Gba1* locus (WT), GCase activity in GD1 mice was undetectable in live blood monocytes, as measured by a flow cytometry-based assay using a membrane-permeable fluorogenic substrate, and was significantly reduced in the serum, confirming enzyme deficiency (Fig. 1b,c; **gating strategy in Supplementary Fig. 2**).

GD1 mice developed marked hepatosplenomegaly by 3 months (Fig. 1d, e). At 12 months, GD1 spleens were tenfold larger than those in Gba^V/V^ mice (2.13% ± 0.54 vs. 0.27% ± 0.10 of body weight, Fig. 1d), and liver weights were nearly doubled (7.9% ± 0.6 vs. 4.1% ± 1.3, Fig. 1e). Even at 12 months, no differences were observed between Gba^V/V^ and WT mice, supporting the use of the parental Gba^V/V^ strain as controls (**Extended data** Fig. 1). Histological analysis revealed extensive infiltration of multinucleated, lipid-laden macrophages (Gaucher cells) in liver and spleen as early as 3 months (**Extended data** Fig. 2). Consistent with the enzymatic deficit, we measured progressive accumulation of disease-associated lipid substrates. Total glucosylsphingosine (GlcSph) levels were markedly elevated in GD1 mice at 3, 6, and 12 months compared to Gba^V/V^ control mice reaching over 80 pmol/mg wet tissue weights by 12 months (Fig. 1f). Similarly, total glucosylceramide (GlcCer) levels and individual GlcCer species were also significantly increased across all time points, exceeding 15,000 pmol/mg by 12 months (Fig. 1g **and Supplementary Fig. 3**). These findings confirmed early and sustained biochemical manifestations of Gaucher disease in this inducible model.

Gaucher disease patients commonly exhibit hematological abnormalities, including anemia and thrombocytopenia, and less frequently, leukopenia [47, 48]. While earlier mouse models required up to 16 months to develop anemia [41], our inducible GD1 model demonstrated earlier onset and more pronounced hematologic pathology. By 3 months of age, GD1 mice exhibited microcytic anemia with significant reductions in hemoglobin (HGB), hematocrit (HCT), and mean corpuscular volume (MCV) (Fig. 1h,i; **Extended data** Fig. 3a-f). At 12 months, HGB, HCT, MCV, and MCH were reduced to approximately 85%, 77%, and 84% of values observed in Gba^V/V^ controls, respectively. Platelet (Plt) and white blood cell (WBC) counts were variable and not significantly different. However, WBC were often elevated in mice at 12 months (**Extended data** Fig. 3g-h). Flow cytometry analysis of peripheral blood cells revealed increased proportions of myeloid CD11b^+^/Ly6C^+^ cells, while mature B (CD19^+^) and T (CD3^+^) cell populations remained unchanged; however, the myeloid expansion was unlikely to fully account for the elevated WBC (**Extended data** Fig. 3i). As reported previously, *Gba*^*V/V*^ mice showed no hematologic differences compared to wild-type mice up to 12 months supporting their use as control mice (**Extended data** Fig. 3).

Bone involvement, a hallmark of GD1, was also more severe and occurred earlier in this model. Whereas prior models exhibited only modest trabecular bone mineral density (BMD) loss by 14 months [42], GD1 mice showed significant femoral BMD reduction by 6 months, consistent with accelerated skeletal pathology (Fig. 1J). In contrast, Gba^V/V^ mice maintained normal BMD compared to wild-type controls (**Extended data** Fig. 3).

In conclusion, our GD1 mouse model recapitulates key visceral, hematologic, and skeletal features of human GD1 disease with significantly faster onset than previous models.

### Low-level chimerism of GCase-expressing cells corrects GD1 phenotypes in vivo

**Figure 2:**
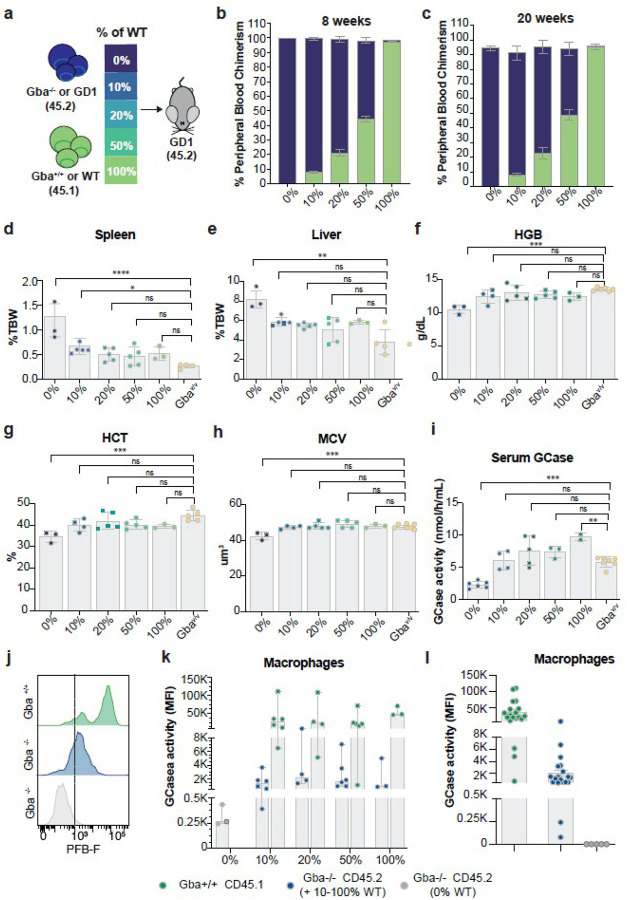
Low-level chimerism of GCase-expressing cells corrects GD1 phenotypes in vivo **a,** Schematic of transplantation experiment. Bone marrow cells from GD1 (Gba^−/−^; CD45.2) and WT (Gba^+/+^; CD45.1) mice were mixed to generate grafts containing 0%, 10%, 20%, 50%, or 100% WT cells. Mice per group: 0% n=4, 10% n=6, 20% n=5, 50% n=5, and 100% n=3. **b–c,** Flow cytometry analysis of peripheral blood showing the relative proportions of WT (CD45.1+, green) and GD1 (CD45.2+, blue) cells at 8- and 20-weeks post-transplant. **d,** Serum GCase activity, expressed as nmol/h/mL, in all transplanted mice containing mixed grafts 0%, 10%, 20%, 50%, and 100% WT cells) as well as a Gba^V/V^ group (yellow) as a control. Each dot represents an individual mouse. **e–f,** Spleen and liver weights expressed as a percentage of total body weight, measured at 5 months post-transplant (corresponding to 8 months of age). **g-i,** Complete blood cell counts for all groups showing hemoglobin (HGB), Hematocrit (HCT), Mean red blood cell volumes (MCV) values. **j,** Representative flow cytometry histograms showing GCase activity (PFB-F) in WT donor, recipient GD1, and control GD1 macrophages. **k,** GCase activity in peritoneal CD45/CD11b+ macrophages measured by flow cytometry. Activity is reported as median fluorescence intensity (MFI) in WT (CD45.1+; green circles) and recipient GD1 (CD45.2+; blue circles) cells. **l,** Background subtracted MFI values of GCase activity in WT donor (CD45.1+; green circles), recipient GD1 (CD45.2+; blue circles) in mice with mixed chimerism, and non-transplanted GD1 controls (CD45.2+; gray circles). Each dot represents data from one mouse. Data are presented as mean ± SD. For **2d, f-i,** statistical analysis was performed using one-way ANOVA with Dunnett’s multiple comparison test. For **2e**, statistical analysis was performed using one-way ANOVA using Sidak’s multiple comparison test. Significant differences are indicated by asterisks (*p < 0.05; **p < 0.01; ***p < 0.001; and ****p < 0.0001).

Genome editing is unlikely to correct all HSCs, which would result in chimeric bone marrow consisting of both corrected and uncorrected cells. To examine how these cells interact in vivo and to determine the minimum proportion of GCase-expressing cells necessary to correct disease manifestations in our GD1 mouse model, we conducted an in vivo dose-escalation study. To achieve this, we transplanted increasing ratios of WT (Gba^+/+^) to GD1 (Gba^−/−^) cells (Fig. 2a). Gba^+/+^ cells producing wild-type GCase levels expressed the CD45.1 isoform, enabling differentiation from the CD45.2-expressing GD1 host cells. Transplants were performed in symptomatic GD1 mice approximately three months after disease induction, following myeloablative total body irradiation.

We assessed biochemical, visceral, and hematological outcomes twenty weeks after transplantation in five groups of mice receiving varying proportions of wild-type (Gba^+/+^) to knockout (Gba^−/−^) HSPCs: 0%, 10%, 20%, 50%, and 100% Gba^+/+^. We also included a sixth group of Gba^V/V^ mice as controls (n = 3–6 mice per group; Fig. 2a). CD45.1/CD45.2 chimerism at eight weeks post-transplant and at study endpoint closely matched the intended input ratios, indicating that Gba^−/−^ HSCs engrafted normally (Fig. 2b and c). Peripheral blood analysis at twenty weeks showed no defects in the multilineage differentiation of Gba^−/−^ cells into mature myeloid, B, or T cells (**Supplementary Fig. 2 and Extended data** Fig. 4). Notably, just 10% Gba^+/+^ cell chimerism was sufficient to normalize hepatomegaly and hematologic parameters, while 20% chimerism fully restored all measured endpoints (Fig. 2d–h **and Extended data** Fig. 5). These findings align with prior studies in GD1 mouse models with wild-type levels GCase expression in non-hematopoietic tissues, showing that a relatively low proportion (~ 7%) of wild-type hematopoietic cells is sufficient to reverse pathology in bone marrow and spleen [49].

To determine the level of circulating GCase activity needed to normalize symptoms of GD1, we measured serum GCase activity in the different transplant cohorts. As expected, mice that received 10% Gba^+/+^ donor cells exhibited serum GCase activity comparable to that of asymptomatic Gba^V/V^ controls (Fig. 2i). We also conducted flow cytometry-based analyses to evaluate intracellular GCase activity in live peritoneal macrophages. Interestingly, Gba^−/−^ macrophages from mice transplanted with wild-type cells consistently showed measurable GCase activity (Fig. 2j–l). However, the enzymatic activity acquired by the knockout cells did not increase proportionally with the level of chimerism. Instead, it plateaued at approximately 6% of the activity observed in wild-type macrophages (Fig. 2l). These findings suggest that there is modest in vivo enzymatic cross-correction occurring between Gba^+/+^ CD45.1 + and Gba^−/−^ CD45.2 + macrophages that lack endogenous GCase activity.

Together, these data support a therapeutic threshold of 10% cells expressing wild-type levels of GCase to normalize visceral and hematological abnormalities in GD1 and demonstrate the ability of GCase-expressing cells to ameliorate disease manifestations in a chimeric hematopoietic system containing corrected and uncorrected cells.

### Efficient expansion and genome editing of GD1 mouse hematopoietic stem and progenitor cells

**Figure 3:**
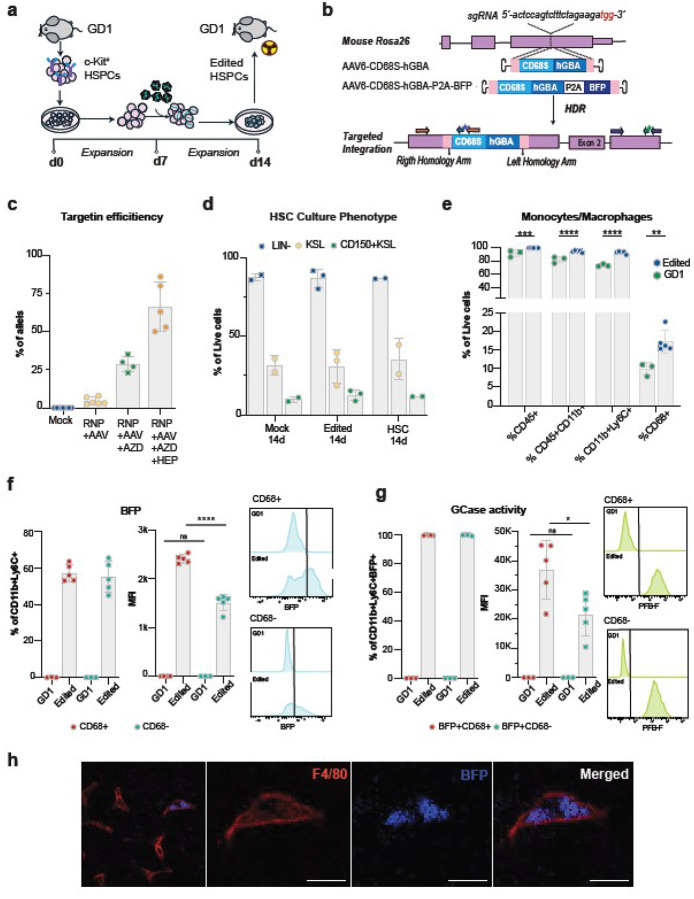
Efficient expansion and genome editing of GD1 mouse hematopoietic stem and progenitor cells. **a,** Schematic of mouse HSPCs isolation, in vitro expansion, and targeting by RNP nucleofection followed by rAAV6 transduction. **b,** Schematic of targeted integration of CD68S-hGBA expression cassettes. The rAAV6 genome was constructed to have 500 bp arms of homology centered on the cut site, and the hGBA sequence was placed under the control of the human CD68 small promoter. Color arrows show the two primer-probe schemes to measure the cassette integration into the *Rosa26* locus by ddPCR. The orange arrows indicate primers, and the blue arrow with the star indicates the probe for the HDR. The purple arrows indicate the set of primers and the green arrow with the star the probe for the reference sequence which lies downstream in the *Rosa26* gene. **c,** Targeting frequencies in GD1 HSPC with and without AZD7648 and HEP HDR enhancers compared to mock HSCPs. Each dot represents an independent targeting assay. For RNP + AAV6 conditions with AZD + HEP, n= 5; AZD, *n* = 4, without *n* = 3, and Mock *n* = 7. **d,** HSC cultures analysis during the expansion and targeting process and transplant day to determine the expression of c-Kit, Sca1, Lineage, and CD150 HSC markers. The bar graph represents the results observed for bulk HSC culture analyzed by flow cytometry. **e,** Mouse CD45, CD11b, Ly6C, and CD68 marker expression in HSPC-derived macrophages after in vitro differentiation, compared to unedited cells. These cells were edited with the rAAV6 containing the CD68S-hGBA-P2A-BFP cassette. **f,** Percentage of BFP-positive cells, BFP mean fluorescence intensity (MFI) and representative histograms in single cells measured by flow cytometry in CD11b+/Ly6C+CD68+ (red) and CD68− cells (aqua). **g,** Percentage of 5-(Pentafluorobenzoylamino)fluorescein (PFB-F) positive cells and PFB-F MFI and representative histograms in single cells measured by flow cytometry using the membrane-permeable substrate PFB-FDGlu (5-(Pentafluorobenzoylamino) Fluorescein Di-beta-D-Glucopyranoside). GCase activity is quantified as PFB-F MFI measured in BFP+CD11b+Ly6C+CD68+ (red) and BFP+CD11b+Ly6C+CD68− cells (aqua). **h,** Representative images of CD45+F4/80+BFP+ macrophages in the liver after transplantation of mHSPCs edited with the CD68S-hGBA-P2A-BFP cassette. Scale bars are 20 μm. All data are presented as mean ± SD. Statistical analysis was two-way ANOVA with Sidak’s multiple comparisons test. Significant differences are indicated by asterisks (*p < 0.05; **p < 0.01; ***p < 0.001; and ****p < 0.0001).

To evaluate the efficacy of ex vivo genome editing in HSPCs as a potential treatment for GD, we developed a protocol for the efficient expansion and targeted editing of mouse HSPCs. We isolated c-Kit^+^ cells from the bone marrow of GD1 mice using immunomagnetic positive selection and cultured them in a medium enriched with a cytokine cocktail that promotes stem cell expansion while preventing differentiation [50] (Fig. 3a). For genome editing, we utilized the RNP-rAAV6 system in which the rAAV6 vector was designed to insert a wild-type human GCase expression cassette into the mouse *Rosa26* safe harbor locus. The expression cassette was driven by a small, synthetic CD68S promoter—a truncated version of the human CD68 promoter—that restricts expression to the monocyte/macrophage lineage, thereby preventing GCase expression in the stem cell compartment and minimizing potential toxicity [35, 51, 52] (Fig. 3b). For in vitro validation, we also created a reporter vector that expressed blue fluorescent protein (BFP) downstream of GCase via a P2A self-cleaving peptide. This design allowed for stoichiometric co-expression of GCase and BFP, facilitating the tracking of edited cells. HDR efficiency was quantified by ddPCR targeting the junction between the 5′ homology arm and the integrated CD68S promoter (Fig. 3b).

Baseline HDR-mediated editing efficiencies in mouse HSPCs were low, averaging 4.5% ± 2.0% (mean ± SD) (Fig. 3c). To enhance HDR, we tested two complementary strategies: suppression of the competing non-homologous end joining (NHEJ) pathway and relief of 53BP1-mediated inhibition of DNA end resection. First, we evaluated AZD7648, a potent and selective inhibitor of the DNA-dependent protein kinase catalytic subunit (DNA-PKcs), a core component of the NHEJ machinery [53, 54]. Inhibition of DNA-dependent protein kinase catalytic subunit (DNA-PKc) with AZD7648 has been shown to enhance HDR by transiently suppressing the competing NHEJ pathway [55, 56]. Treatment with AZD7648 alone increased HDR efficiency to 28% ± 5% (Fig. 3c).

We next assessed HDR Enhancer Protein (HEP), an engineered ubiquitin variant with high affinity for 53BP1, a chromatin-bound factor that suppresses end resection and thereby limits HDR. Inhibition of 53BP1 has been shown to promote HDR by facilitating resection at DNA break sites, a critical prerequisite for homology-dependent repair [57]. Prior studies using a different ubiquitin variant demonstrated increased HDR frequencies in multiple cell types [58–60]. When combined with AZD7648, HEP further increased HDR efficiency to 66.4% ± 16%, indicating a strong synergistic effect from concurrent inhibition of NHEJ and 53BP1-mediated resection blockade (Fig. 3c).

Importantly, edited HSPCs retained phenotypic markers of stem and progenitor identity. Cells were cultured for an additional seven days post-editing, during which flow cytometric analysis of c-Kit, Sca-1, lineage markers, and CD150 revealed enrichment of primitive hematopoietic populations. By day 14, the proportions of Lin^−^, KSL (c-Kit^+^/Sca-1^+^/Lin^−^), and KSL/CD150^+^ cells were comparable across unedited, mock-treated, and edited groups, indicating that genome editing and HDR enhancer exposure did not adversely affect HSPC viability or identity (Fig. 3d).

To evaluate the capacity of edited HSPCs to differentiate into macrophages and test the lineage specificity of the CD68S promoter, cells were cultured in a myeloid differentiation cocktail containing M-CSF and GM-CSF. After 14 days, both edited and unedited GD1-derived HSPCs expressed macrophage-associated markers, including CD11b, Ly6C, and CD68, indicating successful differentiation (Fig. 3e). Interestingly, the proportions of CD11b+, CD11b+/Ly6C+, and CD68^+^ cells were reduced in the unedited group (GD1 cells), suggesting impaired differentiation or reduced proliferation in GCase-deficient cells (Fig. 3e; **gating scheme in Supplementary Fig. 4**).

To assess the activity of the CD68S promoter, we measured BFP expression in HPSCs that were edited using the CD68S-GBA-P2A-BFP cassette after differentiating them into macrophages (Fig. 3f). The percentage of BFP^+^ cells was similar in CD68− and CD68 + populations, approximately 55–57% (left panel). However, the MFI (right panel) was higher in the CD68^+^ subset, indicating promoter activation in these cells upon differentiation. Additionally, an analysis of GCase enzyme activity in the BFP + cells, conducted with the flow cytometry-based enzymatic assay, confirmed GCase activity in the edited GD1 BFP^+^ cells. The GCase levels were found to be approximately 1.7-fold higher in CD68^+^ cells compared to their CD68^−^ counterparts (Fig. 3g).

To assess the in vivo functionality and differentiation of edited HSPCs, we transplanted BFP–reporter–targeted HSPCs into Gba^V/V^ mice. Immunofluorescence analysis of liver sections revealed BFP^+^/F4/80^+^ macrophages, confirming the successful engraftment, differentiation, and tissue integration of gene-edited cells (Fig. 3h).

### Genome-edited HSPCs reverse GD1 pathology following myeloablative conditioning

We investigated whether our genome-editing strategy could reverse or prevent disease manifestations in GD1 mice with established pathology. Three-month-old GD1 mice underwent total body irradiation (TBI, 10 Gy) to achieve complete myeloablation, followed by transplantation with edited HSPCs (referred to as GD1-Treated, n = 6). To control for the effects of the irradiation, we included two additional groups: GD1 mice conditioned and transplanted with unedited GD1 HSPCs (designated as GD1-Sham, n = 6) and control Gba^V/V^ mice transplanted with Gba^V/V^ HSPCs (designated as Gba^V/V^-Sham, n = 5). Twenty weeks after transplantation, we evaluated the mice in all three groups for the engraftment of edited cells, as well as for spleen and liver weights, anemia, and biochemical and histological markers of Gaucher disease.

**Figure 4:**
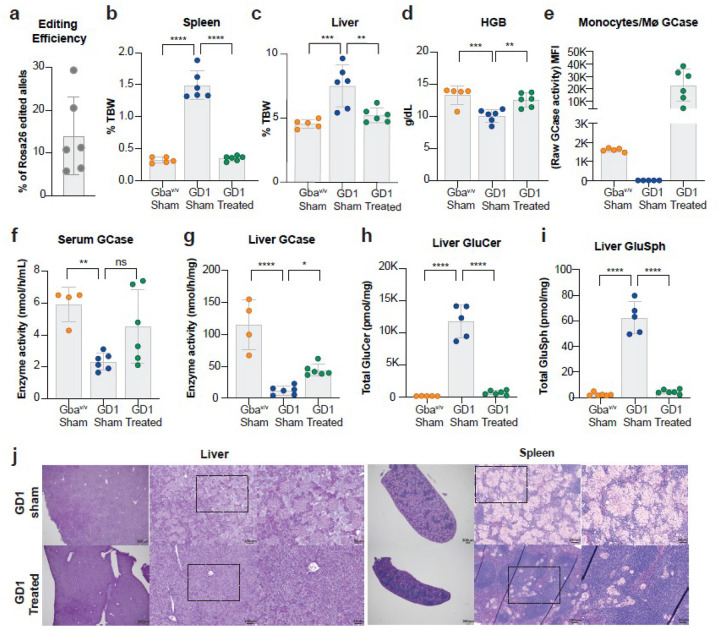
Genome-edited HSPCs reverse GD1 pathology following myeloablative conditioning. The comparison groups included three cohorts: (1) healthy controls consisting of age-matched wild-type mice (Gba^V/V^) that were conditioned with total body radiation (TBI) and transplanted with Gba^V/V^ HSPCs (Gba^V/V^-Sham; *n* = 5); (2) disease controls, which included GD1 mice conditioned and transplanted with unedited GD1 HSPCs (GD1-Sham; *n* = 6); and (3) the treated group, composed of GD1 mice that underwent TBI followed by transplantation with genome-edited mouse HSPCs (GD1-Treated; *n* = 6). **a,** Editing efficiency measured as the fraction of *Rosa26*-edited alleles in the bone marrow 20 weeks post-transplantation in treated GD1 mice. **b-c,** Organomegaly was assessed by spleen and liver weight normalized to total body weight (% TBW). Treated GD1 mice showed significantly reduced organ weights compared to Sham-GD1 controls. **d,** Hemoglobin (HGB) levels in peripheral blood were restored in treated GD1 mice, indicating correction of anemia. **e,** GCase enzyme activity in peritoneal macrophage populations (CD11b^+^Ly6C^+^) measured via flow cytometry using the membrane-permeable substrate PFB-FDGlu. GCase activity is quantified as PFB-F MFI. **f-g,** GCase activity in serum and liver tissue lysate was measured enzymatically using a 4-MU fluorometric assay. **h-i,** Total Glucosylceramide (GlcCer) and glucosylsphingosine (GlcSph) levels in liver tissue expressed as pmol/mg of tissue. **j,** Representative images of Periodic acid–Schiff-stained liver and spleen sections shown at increasing magnifications: 2X (scale bar = 300 μm), 10X (scale bar = 100 μm), and 20X (scale bar = 50 μm). All data are presented as mean ± SD. Statistical analysis for all the graphs was one-way ANOVA with Dunnett’s multiple comparisons test. Significant differences are indicated by asterisks (*p < 0.05; **p < 0.01; ***p < 0.001; and ****p < 0.0001

The mean frequency of genome-edited alleles at the *Rosa26* locus in the bone marrow twenty weeks post-transplantation was 14%, ranging from 6–30%, indicating variable engraftment (Fig. 4a). Despite this variability, all treated mice showed resolution of hepatosplenomegaly, with liver and spleen weights reduced by approximately 80% and 98%, respectively, compared to untreated GD1 controls (Fig. 4b,c). Hematologic analysis revealed normalization of hemoglobin levels in treated mice, comparable to those in Gba^V/V^-Sham controls (Fig. 4e). Additional hematologic parameters—hematocrit, mean corpuscular volume (MCV), and mean corpuscular hemoglobin—were also restored (**Extended data** Fig. 6).

In macrophages (CD11b^+^Ly6C^+^), GCase enzymatic activity was increased to levels approximately 14-fold higher than those in Gba^V/V^-Sham mice, corresponding to 112% of wild-type activity (Fig. 4e; Fig. 1b). Not surprisingly, this robust enzymatic correction was not fully reflected in serum GCase levels. This discrepancy is likely due to multiple factors, including the poor secretion of GCase, rapid denaturation of GCase at neutral pH, the contribution of GCase activity from non-hematopoietic organs, and the inherent limitations of measuring this enzyme in serum [61]. Nevertheless, 50% of treated mice exhibited serum GCase activity comparable to that of Gba^V/V^-Sham controls (Fig. 4f). In the liver, total GCase activity was restored to ~ 31.5% of Gba^V/V^-Sham controls and was increased approximately 3.6-fold compared to GD1-Sham mice (Fig. 4g). This restoration of enzymatic function was accompanied by a > 90% reduction in GlcCer and GlcSph levels, effectively normalizing hepatic lipid content (Fig. 4h–I **and Supplementary Fig. 5**). Histological analysis further confirmed a substantial reduction in Gaucher cell infiltration in both liver and spleen (Fig. 4j).

### Establishing a minimal effective dose for genome-edited HSPCs in GD1 using clinically relevant busulfan conditioning

TBI has broad systemic toxicity and is not clinically applicable for patients with GD1. Additionally, TBI can cause damage to various organs, including the skin, gastrointestinal tract, liver, lungs, bones, brain, and eyes. It may also hinder hematopoietic recovery, impair growth and development, and increase the risk of secondary cancers [62–68]. To improve clinical relevance, we conducted another study that employed a reduced-intensity conditioning regimen based on the chemotherapy agent busulfan. Busulfan is an alkylating agent commonly used for pre-transplant conditioning in clinical trials of ex vivo modified HPSCs for lysosomal storage and hematological disorders [69–74].

**Figure 5:**
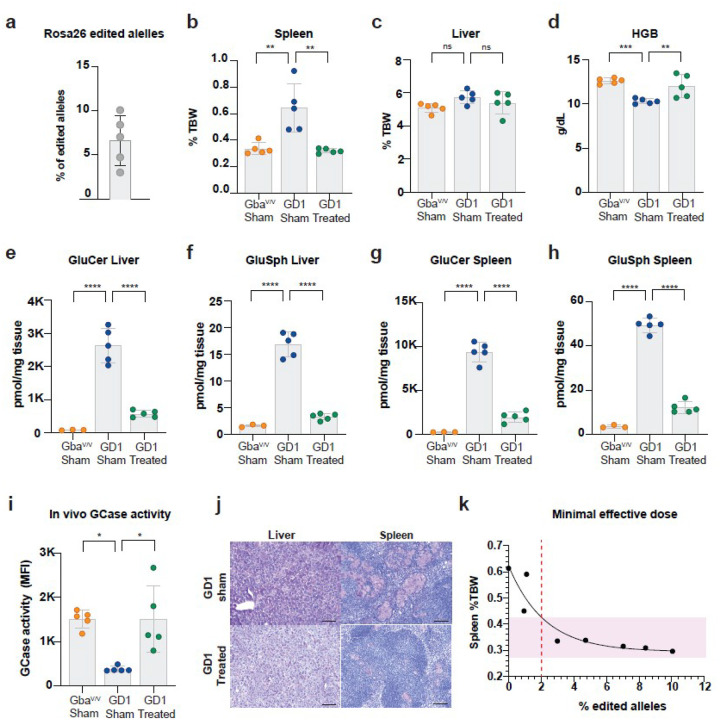
Establishing a minimal effective dose for genome-edited HSPCs in GD1 using clinically relevant busulfan conditioning The comparison groups included three cohorts: (1) healthy controls consisting of age-matched wild-type mice (Gba^V/V^) that were conditioned with busulfan and transplanted with Gba^V/V^ HSPCs (Gba^V/V^-Sham; *n* = 5); (2) disease controls, which included GD1 mice conditioned and transplanted with unedited GD1 HSPCs (GD1-Sham; *n* = 5); and (3) the treated group, composed of GD1 mice that underwent busulfan conditioning followed by transplantation with genome-edited HSPCs (GD1-Treated; *n* = 5). **a,** Fraction of edited *Rosa26* Alleles in bone marrow 16 weeks post-transplantation in GD1-Treated mice. **b-c,** Comparison of spleen and liver weight as a percentage of body weight (%TBW). **d,** Hemoglobin (HGB). **e-h,** Total Glucosylceramide (GlcCer) and glucosylsphingosine (GlcSph) levels in liver (**e and f**) and spleen (**g and h**) expressed as pmol/mg of wet tissue. **i,** Enzyme activity in macrophages measured via flow cytometry using the membrane-permeable substrate PFB-FDGlu. GCase activity is quantified as PFB-F MFI. **j,** Periodic acid-Schiff staining of classical foamy Gaucher cells in two representative sections of the liver and spleen from GD1-Sham and GD1-Treated mice. The sections are representative of a total of 5 sections from 3 independent mice per condition. Scale bars (20X,100um). **k,** Spleen %TBW plotted against the percentage of edited alleles in the bone marrow in all GD1-Treated mice. A nonlinear regression line (black) demonstrates a steep decline in editing efficiency as spleen size increases. The shaded region (0.3–0.4 %TBW) highlights the normal range spleen %TBW observed in healthy mice. The red dashed vertical line marks this inflection point and the minimum threshold of editing required to achieve therapeutic efficacy. All data are presented as mean ±SD. Statistical analysis for all the graphs was one-way ANOVA with Dunnett’s multiple comparisons test. Significant differences are indicated by asterisks (*p < 0.05; **p < 0.01; ***p < 0.001; and ****p < 0.0001.

Two-month-old GD1 mice were conditioned with busulfan administered over four consecutive days and transplanted with GD1 genome-edited HSPCs as previously described. To control for the effects of conditioning, GD1-Sham1 mice and healthy Gba^V/V^-Sham mice were included, both of which received busulfan conditioning followed by autologous HSPC transplantation. The input HSPC population contained 23% edited alleles prior to transplantation. Sixteen weeks post-transplantation, the frequency of genome-edited alleles at the *Rosa26* locus in the bone marrow ranged from 3–10%, with a mean of 6.6% (Fig. 5a), reflecting reduced engraftment likely due to the less intensive myeloablation. Two mice with very low editing frequencies (< 1%) were excluded from downstream analyses.

Despite lower chimerism compared to the TBI-conditioned cohort, GD1-treated mice exhibited phenotypic improvements, including normalized spleen size (Fig. 5b) and correction of anemia, as indicated by hemoglobin levels (Fig. 5d; **Extended data Fig. 7**). Transplantation of genome-edited HSPCs resulted in significant reductions in pathogenic lipids, with total GlcCer and GlcSph levels markedly decreased in both liver (Fig. 5e–f). and spleen (Fig. 5g–h and **Supplementary Fig. 6**). GCase enzymatic activity in peritoneal macrophages was also normalized compared to Gba^V/V^-Sham control with some mice displaying supraphysiological levels (Fig. 5i). Histological analysis confirmed reduced Gaucher cell infiltration in both liver and spleen (Fig. 5j). As expected, the most pronounced therapeutic effects were observed in animals with higher frequencies of edited alleles.

The therapeutic improvement observed following busulfan-based transplantation was less pronounced than that seen in the TBI-conditioned cohorts. One contributing factor was the lower engraftment of genome-edited cells, reflecting the reduced intensity of myeloablation. Additionally, we noticed that GD1-Sham mice in this cohort displayed a significantly milder phenotype than age-matched, unmanipulated GD1 controls, suggesting that busulfan itself may confer partial therapeutic benefit through its myeloablative effects (Fig. 5c **and Extended data Fig. 8**).

We leveraged the lower and more variable engraftment observed in this cohort—including mice with < 1% edited alleles and no phenotypic correction—to refine the minimal effective dose (MED) of genome-edited HSPCs in GD1. We analyzed the relationship between spleen size and the frequency of edited alleles. As shown in Fig. 5k, spleen weight (% total body weight) decreased with increasing levels of genome editing, following a nonlinear regression. A threshold of approximately 2% edited alleles corresponded to the transition into the normal spleen size range, suggesting this value as a candidate MED. Interestingly, even in mice with only approximately 1% edited alleles, although phenotypic improvements in organ size and anemia were not observed at the study endpoint, glucocerebroside accumulation was significantly reduced, indicating partial biochemical correction even with very few corrected cells (**Extended data Fig. 9**).

#### Translation-ready genome editing in human HSPCs achieves high-efficiency CD68S-GBA integration at CCR5

To facilitate the clinical application of our gene-editing strategy for GD, we optimized the HDR-mediated integration of the CD68S-GBA cassette into the human *CCR5* safe harbor locus in adult peripheral blood-derived CD34^+^ HSPCs (PB-CD34^+^ HSPCs). The editing was conducted using the same RNP–rAAV6 delivery platform previously employed for mouse HSPCs. Earlier studies utilizing this system in adult HSPCs reported a low HDR efficiency of approximately 20%. These studies required high doses of rAAV6, which adversely affected long-term stem cell engraftment and resulted in a significant loss of edited alleles between the input and the engrafted cells [35, 36]. To overcome these challenges, we explored two complementary approaches: (1) identifying a more efficient sgRNA and (2) utilizing an HDR enhancer.

**Figure 6:**
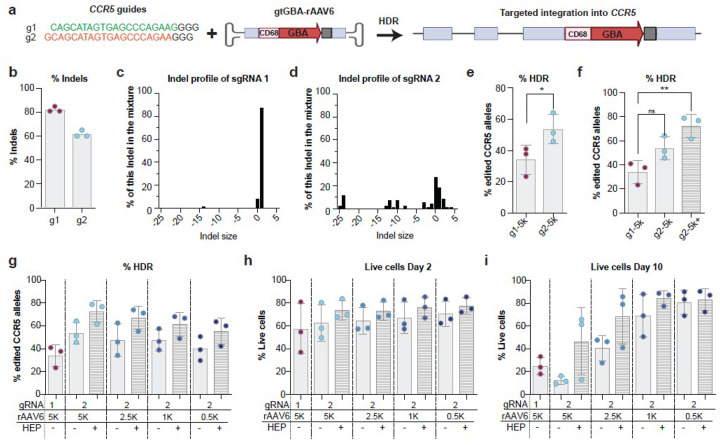
Optimization of sgRNA selection and HDR enhancement for targeted integration of CD68S-GBA into *CCR5*in human PB-CD34^+^ HSPCs. **a,** Schematic showing *CCR5*exon 3 target sites for sgRNAs g1 and g2, which differ by 1 bp, and the AAV6 donor design (*gtGBA-rAAV6)* for targeted integration of the CD68S-GBA cassette using 500 bp homology arms. **b,** Editing efficiency (% indels) of g1 and g2 in PB-CD34^+^ HSPCs, as measured by ICE analysis. **c, d,** Indel profiles for g1 and g2, respectively, showing the distribution and frequency of indel sizes; g2 induces a higher proportion of ≥3 bp deletions consistent with MMEJ repair. **e,**Percent targeted integration (% HDR) comparing g1 and g2 using 5k vg/cell rAAV6 without 53BP1 inhibitor HEP. **f,** HDR efficiency comparing g1 and g2 at 5k vg/cell, with and without HEP. **g,** HDR efficiencies across a range of rAAV6 doses (0.5k–5k vg/cell) using g2, with or without HEP. **h, i,** Short-term (Day 2) and long-term (Day 10) viability of edited PB-CD34^+^ HSPCs, measured as % live cells, across the same rAAV6 titration series. All values represent mean ± SD from n = 3 independent donors. Statistical analysis for **e**, was one-tailed paired t-test and for **f** one-way ANOVA with Dunnett’s multiple comparisons test. Significant differences are indicated by asterisks (*p < 0.05; and **p < 0.01).

We first compared two sgRNAs targeting exon 3 of *CCR5*: g1, which has been used in prior studies, and g2, which cleaves one base pair upstream of g1 (Fig. 6a). Compared to *CCR5* g1, g2 produces lower total indels but a higher proportion of deletions of 3 base pairs or more, with mutational signatures predicted to be associated with microhomology-mediated end joining (MMEJ) and improved HDR outcomes [75] (Fig. 6b–d). The AAV6 donor vector (gtGBA-rAAV6) designed to serve as the template for HDR had the CD68S-GBA cassette between two 500 bp *CCR5* homology arms centered on the *CCR5* g1 cut site. Due to their proximity, both g1 and g2 were compatible with this same HDR template (Fig. 6a). Editing was performed using RNP electroporation and transduction with 5,000 vector genomes per cell (vg/cell) of gtGBA-rAAV6. Across three independent human PB-CD34^+^ HSPC donors, *CCR5* g2 consistently outperformed g1, yielding a 1.6-fold increase in HDR efficiency (Fig. 6e).

We also evaluated HDR enhancers based on prior studies in mouse HSPCs. Although the DNA-PKcs inhibitor AZD7648 improved editing efficiency in combination with guide RNA g1, we excluded it from our human HSPC editing protocol due to concerns regarding potential genotoxicity [76]. Instead, we tested HDR Enhancer Protein (HEP), a high-affinity inhibitor of 53BP1. Importantly, transient 53BP1 inhibition has not been associated with increased translocation frequency or chromosomal instability in CD34^+^ HSPCs, as demonstrated by ddPCR, long-range PCR, and CAST-Seq analyses [58, 60]. Incorporation of HEP into the editing protocol significantly enhanced targeted integration, yielding a 2.3-fold increase in HDR efficiency when using guide g2 compared to g1 alone (Fig. 6f).

We next conducted a titration experiment to evaluate the combined effects of guide selection and HDR enhancement across different doses of the rAAV6 vector. PB-CD34^+^ HSPCs were edited using g2 and transduced with rAAV6 at doses ranging from 0.5K to 5K vg/cell, both with and without HEP. We benchmarked editing efficiency and cell viability against the original condition (g1 + 5K vg/cell without HEP). Notably, even at significantly reduced vector doses g2 outperformed the standard protocol when combined HEP. Specifically, using g2 with HEP at the lowest dose of 0.5K vg/cell achieved 55% targeted integration, which surpassed the performance of g1 at a tenfold higher AAV6 dose (Fig. 6g).

We observed that short-term (day 2) and long-term (day 15) viability decreased with higher AAV6 doses. However, at intermediate and high rAAV6 doses, this viability was consistently preserved, or even improved, with the inclusion of HEP. The lowest dose (0.5K vg/cell) supported robust cell viability, regardless of whether HEP was used (Fig. 6h–i). A direct comparison of g2 with and without HEP confirmed that HEP enhanced editing efficiency and long-term viability.

This work establishes an optimized genome editing protocol for adult human HSPCs that achieves high HDR efficiency and maintains viability at reduced vector doses. The use of *CCR5* g2 and a clinically scalable HDR Enhancer Protein supports the development of safe, effective, and manufacturable autologous HSPC therapies for Gaucher disease.

## Discussion

Our study demonstrates that genome-edited HSPCs engineered to express GCase from a macrophage-specific promoter can effectively reverse the clinical manifestations of GD1 in a novel, rapidly progressive murine model. Using the *Rosa26* safe harbor for targeted insertion of a human CD68S-GBA cassette via CRISPR/Cas9 and rAAV6, we showed that autologous transplantation of edited cells restored hematologic parameters, normalized hepatosplenomegaly, and reduced tissue glucosylceramide and glucosylsphingosine accumulation. Efficacy was observed under both myeloablative and reduced-intensity conditioning, and even low levels of edited allele engraftment led to phenotypic improvements. Additionally, by testing multiple guides we established a highly efficient editing protocol for human HSPC by targeting the same cassette to the human CCR5 locus,

This autologous transplantation strategy presents a promising complementary approach to existing investigational therapies, potentially offering unique benefits, especially when paired with reduced-intensity or non-genotoxic conditioning [77–79]. Non-targeted gene addition into human HSPCs has been explored, using retroviruses and lentiviral vectors [41, 80]. A candidate cell product was tested in humans with promising results [81]. However, ongoing concerns about insertional mutagenesis and the risk of malignant transformation [82] highlight the opportunity for more precise strategies. In vivo, adeno-associated virus (AAV)-based therapies still face challenges related to pre-existing immunity [83], and have raised important safety considerations due to adverse events observed in both clinical and preclinical settings, including death, neurotoxicity, and oncogenic risks such as hepatocellular carcinoma and clonal hepatocyte expansion [84, 85]. Small molecule and recombinant enzyme therapies may be beneficial but are non-curative. In contrast, our ex vivo genome editing platform offers a constructive solution by enabling precise, locus-specific integration that reduces the risks associated with viral vector integration. The ex vivo modification allows for comprehensive product characterization before infusion and is mutation-agnostic. These features position autologous transplantation of ex vivo genome-edited HSPCs as a promising alternative to current therapies for GD1 with potential translational applicability to neuronopathic forms of the disease.

Creating a robust GD1 mouse model has been historically challenging. Early *Gba* knockouts and point mutation models were perinatally lethal or failed to show relevant phenotypes [86, 87]. Conditional models using Mx1-Cre and deletion of exons 9–11 allowed postnatal induction but developed disease features slowly and incompletely [41, 42]. The model described here improves on these by combining Mx1-Cre-mediated excision of exons 6–8 with the D427V Gba1 allele, which reduces residual enzyme activity more effectively than prior configurations. Induction shortly after birth results in early and robust hematopoietic GCase deficiency, leading to hepatosplenomegaly, anemia, skeletal pathology, and lipid accumulation by 3–6 months. This timeline enables rigorous preclinical evaluation of investigational therapies within a clinically relevant therapeutic window. Notably, this model also develops late-onset leukocytosis that cannot be accounted for by expansion of mature blood cell populations, suggesting altered hematopoietic output and highlighting its potential utility for studying the pathogenesis of hematologic malignancies in Gaucher disease [88, 89].

Our studies also established a therapeutic threshold. We demonstrate that as little as 10% chimerism with cells expressing wild-type levels of GCase reverse anemia and hepatosplenomegaly in vivo. This aligns with earlier studies suggesting that low levels of enzymatically competent hematopoietic cells can confer substantial benefit in GD1 [49]. In our busulfan-conditioned cohort, phenotypic correction was observed with only 2–3% editing in the bone marrow, further reinforcing the notion that even partial correction can yield meaningful outcomes. Importantly, the low threshold of correction needed—demonstrated in both wild-type and genome-edited settings—suggests that reduced-intensity conditioning regimens may suffice, improving safety and expanding applicability for patients with GD1. This approach may offer particular benefit for pediatric or high-risk populations and represents a compelling, one-time curative strategy for GD1.

We observed modest but consistent in vivo cross-correction of Gba-null macrophages by donor-derived cells that express GCase. This finding is evidenced by increased intracellular GCase activity in CD45.2^+^ cells that lack the endogenous enzyme. This suggests that even partial chimerism can facilitate meaningful enzyme transfer, thereby enhancing therapeutic benefits. Interestingly, the extent of cross-correction did not increase proportionally with the level of wild-type chimerism, indicating a plateau effect. Notably, unlike most lysosomal enzymes that rely on mannose-6-phosphate receptor (M6PR)-mediated uptake [90], GCase is poorly secreted and instead traffics via Lysosomal integral membrane protein-2 (LIMP-2) [91]. In addition to secretion, alternative mechanisms may also play a role, such as intercellular transfer through cellular connections [92, 93] or delivery via extracellular vesicles [94].

We compared therapeutic outcomes under total body irradiation (TBI) and busulfan conditioning regimens. Under both regimens the edited HSPCs dramatically restored biochemical, hematologic, and visceral manifestations of the disease. As anticipated, TBI conditioning resulted in higher engraftment and more pronounced correction. Nonetheless, busulfan-based conditioning administered at a submaximal dose supported sufficient engraftment of edited cells to yield phenotypic benefit. The slightly lower efficacy observed in the busulfan cohort can be attributed to reduced editing rates and younger age at endpoint, as well as a potential mild therapeutic effect of busulfan itself on disease parameters.

We described an optimized an RNP-rAAV6-based editing platform, achieving highly efficient editing of mouse and human HSPCs. Specifically for human cells, we established a translation-ready genome editing protocol in human peripheral blood-derived CD34^+^ HSPCs that achieves high-efficiency HDR-mediated integration of a CD68S-GBA cassette into the CCR5 safe harbor locus. By identifying a guide with a MMEJ profile sgRNA (CCR5 g2) and incorporating the HDR Enhancer protein, we achieved up to 55% targeted integration using substantially reduced AAV6 vector doses—surpassing the efficiency of prior approaches that required 10-fold higher viral titers. Importantly, these optimized conditions maintained long-term viability and mitigated the cytotoxicity associated with high vector doses. These advances support the clinical scalability of a platform in which genome-edited HSPCs can be engineered ex vivo to express therapeutic levels of GCase in the monocyte/macrophage lineage. Together, the mouse and human datasets provide a coherent preclinical foundation for autologous transplantation of genome-edited cells as a mutation-agnostic, durable therapy for GD1.

## Materials and Methods

### Mice

Mice were housed under a 12:12-h dark/light cycle, temperature (20–22°C), and humidity (30–70%)-controlled environment. Sterile food and water were provided ad libitum in the animal facilities at Stanford University. All experiments were conducted in compliance with the National Institutes of Health institutional guidelines and were approved by the Stanford University Administrative Panel on Laboratory Animal Care (IACUC 33365). Experiments were conducted using male and female mice. At the end of each study, mice were deeply anesthetized with a Ketamine/Xylazine mixture (80 mg/kg Ketamine/16 mg/kg Xylazine, intraperitoneally), exsanguinated and underwent transcardial perfusion with 1X phosphate-buffered saline (PBS-1X, Fisher Scientific 10-010-023).

### Generation of inducible GD1 mouse

GD1 mice were generated by crossing the Gba D427V KI mouse carrying loxP sites flanking exons 6 through 8 of the *Gba1* gene (JAX:019106, Jackson Laboratory (Bar Harbor, ME) with Mx-Cre B6.Cg-Tg(Mx1-cre)1Cgn/J mice (JAX:003556) until obtaining homozygous mice for the D427V *Gba1* and hemizygous the MX1-CRE transgene (*Gba1*^f/f; D427V/D427V^-Tg(Mx1-CRE) or *Gba*-MX). To activate Mx1-Cre in vivo, 750 μg of polyinosinic–polycytidylic acid (pIpC) (Sigma-Aldrich) was injected in three separate doses every second day into pups regardless of genotype (d7, d9, and d11) via intraperitoneal route.

### GD1 Genotyping using droplet digital PCR

Screening of mice after plpC-induced exons deletion was done in peripheral blood after weaning. Genomic DNA was extracted from blood using GeneJET Whole Blood Genomic DNA Purification (Thermo Scientific^™^). For droplet-digital PCR (ddPCR), droplets were generated on a QX200 Droplet Generator (Bio-Rad) per the manufacturer’s protocol. The assay designed to detect excision of *Gba* exons 6–8 consisted of F:5-CCA CAC ATA CAC CTC CCT G −3′, R:5′- GCC ATG CAT CCT TGG CAG-3′, and labeled probe: 5′-FAM/AGGCGCGCC/ZEN/CATCATATGCCTCCATACCT/3IABkFQ/−3′. A HEX reference assay detecting copy number input of the *exon 12* of the *Gba* was used to quantify the chromosome 3 input. The reference assay consisted of: F:5′- AGA TCT TCG GAG GAT GTC CC-3′, R:5′- AAT GTC CAT GCT AAG CCC AG −3′, and labeled probe: 5′– HEX/TCCTGACCT/ZEN/GGGCTTCCTGGAGACCGT/3IABkFQ/−3′. The final concentration of primer and probes was 900 nM and 250 nM, respectively. Twenty microliters of the PCR reaction were used for droplet generation, and 40 μL of the droplets was used in the following PCR conditions: 95° C −10 min with 2° C /s ramp, 50 cycles of 94° C −30 s with 2° C /s ramp, 61°C–30 s, and 72°–2 min with 2° C /s ramp, finalize with 98°–10 min and 4 °C until droplet analysis. Droplets were analyzed on a QX200 Droplet Reader (Bio-Rad), detecting FAM and HEX-positive droplets. Control samples with no genomic DNA and from uninduced mice were included. Data was analyzed using QuantaSoft (Bio-Rad).

### Mouse HSPC isolation and expansion

Lineage negative (Lin−) KIT + SCA-1+ (LKS) HSPCs were isolated from young GD1 mice and cultured based on a published method [50]. Briefly, KIT+ cells were purified from total bone marrow using anti-KIT/CD117 microbeads and following the manufacturer’s instructions (Miltenyi Biotec 130-097-146). Purified KIT+ cells were plated in cell bind plates (Costar 3337) at 5.5 × 10^5^ cells/mL and cultivated for 14 days in F12 media (Gibco 11765–054) supplemented with 100 ng/mL mouse TPO (Peprotech 315–14), 10 ng/mL mouse SCF (Peprotech 250–03), 0.1% Polyvinyl alcohol (PVA, Sigma-Aldrich P8136), 1% HEPES (Gibco 15630–080), 1% ITS-X (Gibco 51500–056) and 1% Penicillin-Streptomycin-Glutamine (Gibco 10378–016). LKS HSPCs were maintained at 37 °C, 5% CO_2_ and 5% O_2,_ half media changes were performed thrice a week. Bulk LKS HSPCs (5.5 × 10^5^ cells/mouse) were transplanted at culture day 14 into 8-week-old female C57BL/6 J mice (Jax strain #000664). Flow cytometry was used to evaluate the fraction of LKS HSPCs in culture at day 7, 11, and 14 using the following antibodies: anti-mouse CD45 PE-Cy7 (clone 30F11 Biolegend), anti-mouse SCA-1/Ly-6A/E PE (clone D7 Thermo Fisher Scientific), anti-mouse c-KIT/CD117 APC (clone 2B8 Thermo Fisher Scientific), and anti-mouse CD150 PE/Cyanine7 antibody (BioLegend, clone TC15–12F12.2).

### Culturing of human HSPCs

Human CD34+ HSPCs mobilized from peripheral blood were purchased frozen from AllCells (Almeda, CA, USA) and thawed per manufacturer’s instructions. Cells were cultured in CellGenix GMP SCGM medium (CellGenix) supplemented with stem cell factor (SCF; 100 ng/mL, PeproTech 300–07), thrombopoietin (TPO; 100 ng/mL, PeproTech 300–18), Flt3-ligand (FTL3, 100 ng/mL, PeproTech 300–19), UM171 (35 nM, STEMCELL Technologies 72914), and 1% penicillin/streptomycin (P/S) under hypoxic conditions.

### rAAV vector plasmid construction

The Rosa26 donor vectors have been constructed by PCR amplifying 500 bp left and right homology arms of the Rosa26 locus from mouse DNA flanking guide RNA region at exon 1. Human wild-type GBA sequences were amplified from plasmids. The CD68S sequence was obtained from Dahl et al [80, 95]. and was cloned from a gblock Gene Fragment (IDT, San Jose, CA, USA). Primers were designed using an online assembly tool (NEBuilder, New England Biolabs, Ipswich, MA, USA) and were ordered from Integrated DNA Technologies (IDT, San Jose, CA, USA). Fragments were Gibson-assembled into a the pAAV-MCS plasmid (Agilent Technologies, Santa Clara, CA, USA). Constructs were planned, visualized, and documented using Snapgene 5 Software. The CCR5 donor (gtGBA-rAAV6) was were similarly constructed except the constructed left and right homology arms were constructed by PCR amplification of 500 bp s from human genomic DNA

### rAAV production

rAAV was produced using a dual-plasmid system as described in Khan et al [96]. Briefly, HEK293 cells were transfected with plasmids encoding an AAV vector and AAV rep and cap genes. HEK293 cells were harvested 48-h post-transfection and lysed using three cycles of freeze-thaw. Cellular debris was pelleted by centrifugation at 1350 × g for 20 min and the supernatant collected. Active rAAV particles were purified using AAVpro purification Kit Midi (Cat#6675), Takara Bio), and stored in PBS at −80 °C. rAAV vectors for in vivo applications were ordered from Vigene Biosciences (Rockville, MD, USA). Viral titers were determined using droplet digital PCR with the following primer/probe combination: F: GGA ACC CCT AGT GAT GGA GTT, R: CGG CCT CAG TGA GCG A, P: /56FAM/CAC TCC CTC/ZEN/TCT GCG CGC TCG/ 3IABkFQ/.

### Genome editing of HSPCs

To target the hCD68S-hGBA cassette into the murine *Rosa26* and human *CCR5* loci, we used Alt-R^®^ S.p. HiFi Cas9 Nuclease V3 (IDT, 1081061) and chemically modified sgRNAs (TriLink BioTechnologies) containing 2′-O-methyl and 3′-phosphorothioate modifications at the three terminal bases to enhance stability and performance.

For mouse genome editing, lineage-negative CD117^+^ HSPCs were isolated and cultured for 7 days prior to electroporation. RNP complexes (30 μg Cas9, 15 μg sgRNA per 1 × 10^6^ cells in 100 μL) were delivered using the Lonza 4D Nucleofector (pulse code OZ100). Immediately post-electroporation, cells were transferred into pre-warmed HSPC culture medium at 37 °C. For *Rosa26* targeting, rAAV6 donor vectors were added at 5,000 vector genomes (vg)/cell.

For human genome editing, CD34^+^ HSPCs were edited on day 2 of culture using the previously described rAAV6-CD68-GBA donor vector [35]. CCR5-g11 and CCR5-g3 sgRNA sequences were 5′-GCAGCATAGTGAGCCCAGAA-3′ and 5′-CAGCATAGTGAGCCCAGAAG-3′, respectively. RNPs were formed at a 1:2.5 molar ratio of Cas9 to sgRNA. Electroporation was performed using the Lonza 4D Nucleofector with program DZ-100 in P3 primary cell solution. For small-scale experiments, 1 × 10^6^ cells were electroporated in 20 μL with 6 μg Cas9 and 3.2 μg sgRNA. For large-scale experiments, up to 5 × 10^6^ cells were electroporated in 100 μL with 30 μg Cas9 and 15 μg sgRNA. Cells were then transferred to pre-warmed culture medium and transduced with rAAV6 donor at 500–5,000 vg/cell.

HDR enhancer compounds were used to improve editing outcomes. Alt-R^™^ HDR Enhancer Protein (HEP; IDT, Cat. #576746828, 4 mM stock in DMSO) was added directly to the electroporation solution at a final concentration of 50 μM. AZD7648 (Selleck Chemicals, S8843) was added to the post-electroporation culture medium at 0.5 μM and removed after 12 hours by media replacement.

### Homology Direct Repair (HDR) quantification with droplet digital PCR

Genomic DNA was extracted from post-edited HSPCswith QuickExtract DNA Extraction Solution. Droplet-digital PCR (ddPCR) was performed as described before with modifications. For the Rosa26 locus, the assay designed to detect insertion of the cassette into the mouse endogenous *Rosa26* consisted of F:5-TCG TCG TCT GAT TGG CTC TC-3′, at the exon 1 of *Rosa26* and R:5′-ACA GTG TCA CAT GGG TGC AA −3′, at the human CD68s promoter. and labeled probe: 5′-FAM/CCCAGAAAA/ZEN/CTGGCCCTTGC/3IABkFQ-3′. The reference assay designed to detect the *Rosa26* intron two sequences: F:5′- AAG CCA CTG ACT ATG GTG CC −3′, R:5′-CTCAGAAGCAGAAGCATCCCT CTCAGAAGCAGAAGCATCCCT-3′ and labeled probe: 5′-HEX/TGTTCTCAA/ZEN/AGGAAGGATTGTCTGTGC/3IABkFQ-3′.

For the human CCR5 locus, the ratio of detected *CCLR2/CCR5* events gave the fraction of targeted alleles in the original cell population. The *CCR5* detection assay was designed as follows: F:5 - GGG AGG ATT GGG AAG ACA-3, R: 5 -AGG TGT TCA GGA GAA GGA CA-3, labeled probe: 5 - FAM/AGC AGG CAT/ZEN/GCT GGG GAT GCG GTG G/3IABkFQ-3. The reference assay was designed as follows: F:5 - CCT CCT GGC TGA GAA AAA G-3, R: 5 - CCT CCT GGC TGA GAA AAA G-3, and probe: /5HEX/TGT TTC CTC/ZEN/CAG GAT AAG GCA GCT GT/3IABkFQ/. For all assays, the final concentration of primer and probes was 900 nM and 250 nM, respectively. Twenty microliters of the PCR reaction were used for droplet generation, and 40 μL of the droplets was used in the following PCR conditions: 95° C −10 min, 50 cycles of 94° C −30 s, 60°C–30 s, and 72°–1 min, finalize with 98°–10 min and 4 °C until droplet analysis. Droplets were analyzed on a QX200 Droplet Reader (Bio-Rad), detecting FAM and HEX-positive droplets. Control samples with no genomic DNA and from uninduced mice were included. Data was analyzed using QuantaSoft (Bio-Rad).

### Transplantation and Conditioning Regimens

Recipient mice were conditioned with either 10 Gy total body irradiation (TBI) or busulfan (Sigma-Aldrich, Cat# 14843). busulfan was administered intraperitoneally at 25 mg/kg/day for four consecutive days (total dose: 100 mg/kg), following standard myeloablation protocols. Transplants were performed 24 hours after the final conditioning dose via retro-orbital sinus injection. Donor bone marrow was harvested from GD1 or CD45.1 congenic wild-type (WT) mice. Whole bone marrow was collected by flushing femurs and tibiae with 1× PBS (Fisher Scientific, Cat# 10-010-023) supplemented with 4 U/mL heparin (Sigma-Aldrich, Cat# H3149–500KU). Cell suspensions were filtered through a 30 μm strainer, washed twice with 1× PBS, and resuspended at 1.5 × 10^8^ cells/mL. To assess the impact of donor chimerism, GD1 and WT bone marrow cells were mixed to achieve final graft compositions containing 0%, 10%, 20%, 50%, or 100% WT cells. Recipient GD1 mice received 10 cGy of irradiation and were transplanted with 2 × 10^6^ total bone marrow cells per mouse (n = 3–6 per group). For experiments involving edited or unedited HSPCs, 5 × 10^5^ cells were transplanted per mouse.

### In vitro Macrophage differentiation and flow cytometry

Mouse HSPCs were seeded at a density of 2 × 10^5^ cells/ml in non-treated 6-well plates containing differentiation medium (DMEM-F12 supplemented with M-CSF [50 ng/ml], GM-CSF [25 ng/ml], and penicillin/streptomycin [10 U/ml]). After 48 hours, non-adherent cells were removed, and adherent cells were kept in the original wells and medium were change every 2 days. After two weeks, adherent macrophages were harvested by incubating with 10 mM EDTA in PBS. For in vivo enzyme activity assays, 2 × 10^5^ cells per condition were incubated with PFB-FDGlu, following the previously described procedure. After substrate incubation, cells were resuspended in 100 μl of staining buffer (PBS with 2% FBS and 0.4% EDTA). To block non-specific antibody binding, 5% (v/v) TruStain FcX (BioLegend, #422302) was added. Cells were then stained with 2 μl of each fluorophore-conjugated monoclonal antibody for 30 minutes at 4 °C in the dark, using the following antibodies: CD45.2-PeCy7 (Invitrogen, 25-0454-82), CD11b-PE (BioLegend, 17-0032-82), Ly-6C-BV605 (BD Horizon, 563011), and CD68-APC (BioLegend, 137008). Propidium iodide (1 μg/ml) was used to identify dead cells. Samples were analyzed using a BD FACSymphony A5 flow cytometer. Flow cytometry data were analyzed using FlowJo software (FlowJo, LLC)

### Flow cytometry analyses of cells isolated from mouse hematopoietic tissues

Mice were euthanized at designated time points for the analysis of donor chimerism in various tissues. Deep anesthesia was induced using a Ketamine/Xylazine mixture (80 mg/kg Ketamine/16 mg/kg Xylazine, intraperitoneally). Following transcardial perfusion with PBS-1X, tibiae, femurs, spleen, Liver, and brain were harvested. Bone marrow cells (BM, from tibiae) were isolated in RPMI (Thermo Fisher Scientific 61870127) supplemented with 10% FBS, 4U/mL Heparin (Sigma Aldrich H3149–500KU) and 0.2 U/mL Deoxyribonuclease I (Worthington Biochemical Corporation LS002007) and filtered through a 30 μm cell strainer. Erythrocytes were lysed using the RBC lysis buffer (Thermo Fisher Scientific 00-4333-57). Afterward, the cells were washed, resuspended in MACS buffer, and maintained on ice until further processing. For flow cytometry staining, cells were blocked for 10 minutes with 10% vol/vol Mouse BD Fc Block^™^ (clone 2.4G2 BD Biosciences) and then stained in the dark for 30 min using the following antibodies: anti-mouse Ter119-Pe-Cy5 (INVITROGEN 15-5921-83); anti-mouse CD45.2-PeCy7 (INVITROGEN 25-0454-82); anti-mouse CD45.1-APC-Cy7 (INVITROGEN 110716); anti-mouse/Human CD11b-PE (Biolegend 17-0032-82); anti-mouse Ly-6C-BV605 (BD Horizon 563011) CD3 APC (INVITROGEN 17-0032-82); anti-mouse CD19-BV650 (BD Horizon 563235), and dead cells were stained using Propidium iodide (PI) counterstain. The cells were then washed and resuspended in MACS buffer. Stained cells were acquired using a BD FACSAria II cell sorter and conducted with BD FACSDiva software. Flow cytometry data were analyzed using FlowJo software (FlowJo, LLC).

### Hematological analysis

Total Cell Blood Count was performed on a XN-1000 automated hematology analyzer (Sysmex America) in 150 uL of EDTA-anticoagulated whole blood in the first 3 hours after collection.

### Histological analyses

Tissue samples were collected following transcardial perfusion with cold PBS-1X, then fixed overnight in 4% paraformaldehyde solution in PBS (Santa Cruz Biotechnology sc-281692). After fixation tissues were embedded in paraffin, and sectioned. Sectioned tissue was stained with periodic acid Schiff for microscopic examination. Images were taken in a BZ-X800 all-in-one fluorescence microscope and BZ-X800 software (Keyence, Itasca).

### Monocyte/Macrophages in vivo enzyme activity

In vivo GCase activity was assessed in peritoneal macrophages collected from different mouse cohorts, following a published protocol [97]. Briefly, peritoneal macrophages were harvested using cold PBS from anesthetized mice prior to euthanasia and treated with RBC lysis buffer. Approximately 5 × 10^5^ cells per sample were plated into two wells of a low-binding 96-well plate. One well (well 1) was treated with 2 μL of 50 mM CBE (Cayman Chemicals, 6090-95-5) to achieve a final concentration of 1 mM, gently mixed, and incubated at 37°C with 5% CO_2_ for 60 minutes. Both wells then received 2 μL of 37.5 mM PFB-FDGlu (final concentration 0.75 mM), mixed, and incubated for an additional 30 minutes under the same conditions. The reaction was terminated by adding 1 ml of ice-cold MACS buffer, followed by centrifugation (300 × g, 5 min, 4 °C) and subsequent cell staining for flow cytometry analysis using the following Antibodies: mTer119-Pe-Cy5 (INVITROGEN 15-5921-83); CD45.2-PeCy7 (INVITROGEN 25-0454-82); CD11b-PE (Biolegend 17-0032-82); Ly-6C-BV605 (BD Horizon 563011). PFB-FDGlu was detected in the FITC channel, and dead cells were gated out using Propidium iodide (PI) counterstain.

### Serum and tissue enzyme activity

GCase activity was determined fluorometrically after 2 h of incubation at 37°C with 5.5 mM 4methylumbelliferyl-d-glucuronide trihydrate (Sigma–Aldrich). Specifically, protein was extracted by lysing cells in 200 μl of deionized water with a Branson Sonicator with probe, centrifuging lysates at 17,000 × *g* for 10 min at 4 °C, and collecting the supernatant containing the soluble proteins. GCase activity in plasma and tissue homogenates was measured using a fluorogenic enzymatic assay. Briefly, 5 μl of each sample was mixed with 15 μl of 4-methylumbelliferyl-β-D-glucopyranoside substrate (Sigma, #M3633) at a final concentration of 5 mM in citrate/phosphate buffer (pH 5.5) containing 15% (w/v) sodium taurocholate. Samples were incubated at 37 °C for 1 hour, protected from light. The reaction was stopped by adding 180 μl of stop buffer (0.2 M glycine/carbonate, pH 10.7). Fluorescence from 4-methylumbelliferone (4MU), released by GCase activity, was measured using a SpectraMax M3 microplate reader (Molecular Devices) with SoftMax Pro 7 software, at 355 nm excitation and 460 nm emission. A 4MU standard curve was prepared using 4MU sodium salt (Sigma) in assay buffer. Protein concentration in the supernatants was determined using the Pierce BCA Protein Assay Kit (Thermo Scientific).

### Glucosylceramide and glucosylsphingosine quantification

For evaluation of glucocerebrosides substrate accumulation, tissue pieces were submerged in liquid nitrogen directly after removal from the mice. Extractions of glucocerebrosides were as described [98]. Following methanol/chloroform/water (2:1:0.6, v/v/v) extraction, the extracts from frozen tissues were subjected to KOH alkaline methanolysis (0.3 N, 0.6 mL) at 37°C (1h) to remove potentially interfering glycolipids. After cooling, approximately 50 μL of 3N HCl was added to neutralize the solution, followed by water (50 μL) and chloroform (1.2 mL). To remove non-lipid contaminants, the samples were brought to 5 mL with chloroform:methanol:water (60/30/4.5, v/v/v) and loaded onto a Sephadex G-25 Fine column equilibrated in the above solvent. The column was washed with 5 mL of chloroform:methanol (2:1, v/v). Sphingolipids were collected (10 mL) and dried under nitrogen gas.

Analyses and quantitation of glucosylceramide and glucosylsphingosine levels were carried out by ultra-high performance liquid chromatography coupled to electrospray tandem mass spectrometry (UPLC-ESI-MS/MS) using a Waters Quattro Micro TQ-S triple quadrupole mass spectrometer (Waters, Milford, MA). The extracted tissues samples were suspended in methanol containing the internal standard and 10 μL was injected for LC-ESI-MS/MS measurements. Chromatographic separation for GlcCer and GlcSph was achieved using a XSelect CSH C18 XP Column (2.1 mm ×100 mm, 2.5 μm, Waters) column. Gradient elution with a mobile phase of acetonitrile and water charged with ammonium formate and formic acid was employed for the separation of sphingolipid species of varying acyl chain length. Nitrogen was used as nebulizer and argon was used as collision gas. The source temperature was maintained at 120 °C, and the desolvation temperature was kept at 425 °C. The drying gas (N_2_) was maintained at ca. 800 L/h, and the cone flow gas was turned off. The multiplier was set at an absolute value of 650 V. Optimized parameters for GlcCer and GlcSph were determined with infusing individual standard compounds (Matreya, LLC and Avanti Polar lipids, Inc.) in MS. Quantification by LC-MS/MS was operated in the multiple reaction monitoring (MRM) mode, with detection of the transition pair of the individual protonated parent ions of GlcCer and daughter ion m/z 264. GlcSph was measured by monitoring of the mass transition m/z 462.3> 282.4. Calibration curves were prepared for C16 GlcCer, C18 GlcCer, C24 GlcCer, and C24:1 GlcCer using C18 glucosyl(ß) ceramide-d5 as the internal standard. Quantification of GlcCer species with various fatty acid chain lengths was established from the calibrations curve of each species or with the closest fatty acyl chain length. The quantification of GlcSph was based on the calibration curve using glucosyl(ß) sphingosine-d5 as internal standard. The calibration curve for both GlcCers and GlcSph was 25 pg–10 ng on column.

The GlcCer species and GlcSph levels were normalized to wet tissue weight (mg). Age-matched control mice tissues were analyzed in parallel with GD1 mouse samples.

### Bone density measurement

Soft tissue was removed from mice femurs and were wrapped with saline-soaked gauze and stored at −20C until analysis. On the day of the analysis, femurs were thawed for 30 minutes at room temperature, and each pair was fitted into a 0.7 mL Eppendorf tube and filled with Dionized water avoiding any bubbles on the inside. The tubes were attached to a multimodal animal cradle and placed inside the micro-CT scanner. The distal end of the femurs was scanned using a Bruker Skyscan 1276 micro CT scanner (x-ray voltage 85 kV, 1 mm aluminum filter; isotropic voxel size 5 μm). Two phantoms of 0.25 and 0.75 g.cm-3 CaHA were also scanned under the same conditions and settings as a control for calibration. 3D Images were reconstructed using SkyScan’s volumetric NRecon reconstruction software, and the bone density analysis was performed in Bruker CTAn Micro-CT Software. Mineral density of the trabecular region of the femur was measured after establishing a region of interest of a volume of 300 slides, starting at 50 slices distal to the growth plate. Bone mineral density (BMD) was calculated by the software and defined as the volumetric density of calcium hydroxyapatite (CaHA) in terms of g.cm-3. It was calibrated by means of phantoms with known densities of 0.25 and 0.75 g.cm-3 CaHA.

### Statistical analyses

All the data presented in this manuscript are expressed as the mean ± standard deviation of the mean (SD). The number of samples, denoted as n, refers to the individual mouse for in vivo experiments (where n=1 per mouse) or to the number of independent biological replicates for in vitro experiments (where one independent biological replicate corresponds to n=1). Statistical analyses were performed using GraphPad Prism 7 (GraphPad Software). Parametric tests were applied to data that followed a normal distribution, as assessed using the Shapiro-Wilk test. The following statistical analyses were as follows: two-tailed unpaired t-test for comparison between two groups, one-way ANOVA with Tukey post hoc or Kruskal-Wallis test with Dunn’s correction for comparisons involving more than two groups, or two-way ANOVA with Tukey’s or Sidak’s post hoc tests for comparisons with multiple variables. The significance threshold for all parametric tests was set at alpha = 0.05, with all tests being two-sided. A p-value of less than 0.05 was considered statistically significant. The specific statistical tests applied to each data set are described in the figure legends. In all figures, significance is denoted as *p < 0.05, **p < 0.01, ***p < 0.001, and ****p < 0.0001. The exact p-values for each comparison are provided in the Source Data file.

## Supplementary Material

Supplementary Files

This is a list of supplementary files associated with this preprint. Click to download.

- SupplementaryFigures.pdf
- PimentelExtendedData.pdf
